# Integrated Laser Additive Manufacturing of α-Al_2_O_3_ Nanoparticle-Seeded β/γ’ Ni-Al Intermetallic Alloy with Enhanced High-Temperature Oxidation Performance

**DOI:** 10.3390/ma16227205

**Published:** 2023-11-17

**Authors:** Xun He, Xiaoyong Shu, Ziyi Zhou, Shouhua Yang, Limei You, Xiao Peng

**Affiliations:** 1 School of Materials Science and Engineering, Nanchang Hangkong University, Nanchang 330063, China; 71053@nchu.edu.cn (X.H.); 70394@nchu.edu.cn (X.S.); zhou.zy@hotmail.com (Z.Z.); by2201158@buaa.edu.cn (S.Y.); 71066@nchu.edu.cn (L.Y.); 2 Jiangxi Provincial Engineering Research Center for Surface Technology of Aeronautical Materials, Nanchang Hangkong University, Nanchang 330063, China; 3 School of Materials Science and Engineering, Beihang University, Beijing 100191, China

**Keywords:** oxidation, laser deposition, intermetallic compounds, aluminum oxide, surface modification

## Abstract

The oxidation of β-NiAl at high temperatures leads to the preferential formation of metastable alumina, such as θ-Al_2_O_3_, which exhibits a significantly faster growth rate compared to stable α-Al_2_O_3_. However, our recent research has shown that through the use of the surface-dispersing nanoparticles (NPs) of metal oxides with a hexagonal closed pack (hcp), such as α-Al_2_O_3_, the thermal growth of α-Al_2_O_3_ can be facilitated. The present study employed laser additive manufacturing (LAM) to develop an integrated α-Al_2_O_3_ NPs surface-seeded two-phase intermetallic alloy comprising brittle β-NiAl and tougher γ’-Ni_3_Al, which demonstrated better comprehensive mechanical properties. It was found that seeding the α-Al_2_O_3_ NPs promoted the early stage growth of α-Al_2_O_3_ on both β and γ’ phases during oxidation in air at 1000 °C. This led to a decrease in the oxidation rate but an enhancement in adhesion of the formed alumina scale in comparison to the naked β/γ’ two-phase alloy. The reasons for this result were interpreted.

## 1. Introduction

The β-NiAl intermetallic compound is a highly valuable high-temperature material with the following merits: low density, high melting point and great thermal conductivity. Unfortunately, the poor strength of B2-structured β-NiAl is one factor limiting its practical use as a high-temperature structural material. γ’-Ni_3_Al exhibits the above attractive properties similar to β-NiAl, but its ordered L1_2_ structure leads to better high-temperature toughness [1,2,3]. Thus, γ’-Ni_3_Al is an indispensable component of β-NiAl, functioning as a toughening agent. The combination of these two nickel aluminide intermetallics is widely utilized as a high-temperature structural material for aircraft engines and aerospace applications [4]. The mechanical performance of β-NiAl/γ’-Ni_3_Al two-phase intermetallic compounds at high temperatures has been extensively investigated [5,6,7,8,9,10,11,12]. Additionally, they have been developed as protective coatings against high-temperature oxidation [13,14]. β/γ’ dual-phase intermetallic alloys were mostly manufactured through the use of directional solidification [5,6,8] and vacuum arc melting methods [7,9,10,11,12].

Laser additive manufacturing (LAM) is a promising near-net-shape-forming technology that can accurately produce complex components via a stacking method of point-by-point, line-by-line and layer-by-layer under the control of a computer-aided design (CAD) system [15,16,17,18]. The laser 3D printing of Ni-Al intermetallic alloys has been reported, with the focus mostly on their mechanical properties [19,20,21,22,23,24]. In this contribution, LAM was applied to manufacture a β/γ’ two-phase Ni-Al intermetallic alloy with better resistance to high-temperature oxidation.

As is known, β-NiAl and γ’-Ni_3_Al are both oxidation-resistant because they are able to form a protective Al_2_O_3_ scale. However, the thermal growth of Al_2_O_3_ scales on the two intermetallic phases follows various processes. In general, γ’-Ni_3_Al cannot grow Al_2_O_3_ until a non-protective NiO layer has been developed [25,26]. At high temperatures, β-NiAl preferentially grows metastable θ-Al_2_O_3_, and it takes time to transform to stable α-Al_2_O_3_ [27,28,29,30,31,32,33,34]. θ-Al_2_O_3_ grows one or two orders of magnitude faster than α-Al_2_O_3_ [29]. Slower θ-to-α transformation normally leads to considerable alumina volume shrinkage and, consequently, micro-crack generation on the alumina scale [29,31,32,33,34]. Recently, Peng and co-workers [35,36] have reported that θ-to-α alumina transformation is bypassed in δ-Ni_2_Al_3_ (which quickly transforms to β-NiAl at high temperatures [37]) by dispersing nanoparticles (NPs) of metal oxides with a hcp structure like α-Al_2_O_3_. This is because the dispersed α-Al_2_O_3_ NPs energetically promoted direct thermal growth of α-Al_2_O_3_ on the nickel aluminide. They also found that the dispersion of α-Al_2_O_3_ NPs onto a Ni-Cr binary alloy can thermodynamically catalyze the growth of iso-structural Cr_2_O_3_, converting the alloy from oxidation-nonresistant to oxidation-resistant [38].

Based on the results above, a β/γ’ two-phase Ni-Al intermetallic alloy seeded with α-Al_2_O_3_ NPs was integratedly manufactured through the use of LAM. The purpose of seeding α-Al_2_O_3_ NPs was to improve the oxidation performance of LAM β/γ’ Ni-Al intermetallic by promoting the thermal growth of α-Al_2_O_3_ in terms of not only the β phase but also the γ’ phase.

## 2. Materials and Methods

### 2.1. Materials

**Figure 1:**
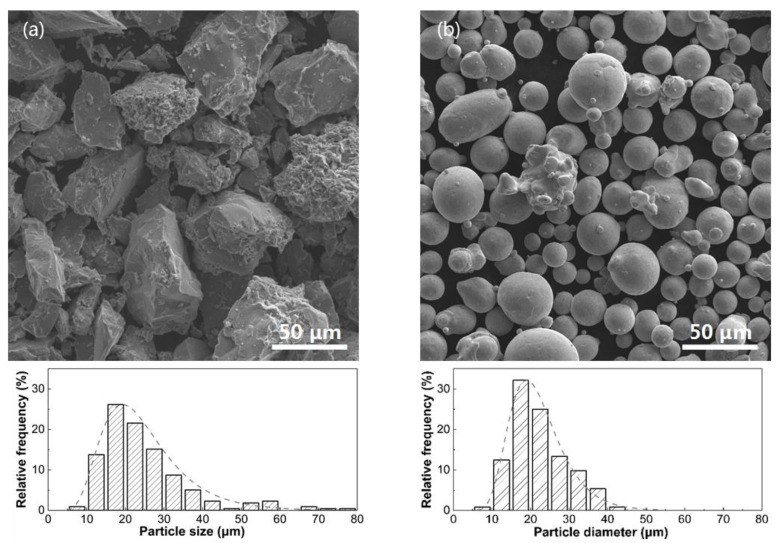
SEM morphologies with their particle size distributions of commercial (**a**) Ni-50Al and (**b**) pure Ni powders.

Laser additively manufacturing the β/γ’ two-phase Ni-Al intermetallic alloy utilized Ni-50Al particles (in weight percentage) crashed from pre-alloyed material and Ni particles (purity > 99.9%) produced through the use of the gas atomization method. The surface morphologies of both particle powders and their sizes were measured using SEM. As depicted in (Figure 1a), the Ni-50Al powder exhibits an irregular gravel-like shape and is sized in the range of ~10 μm to 45 μm, with a mean value of 19.6 μm, as determined by analyzing over 500 particles using ImageJ 1.35t software. The Ni powder appears predominantly spherical and distributed in diameter ranging from ~10 to 80 μm, with a mean value of 19.2 μm, as depicted in Figure 1b.

### 2.2. Methodology to Design β/γ’ Ni-Al Intermetallic Alloy

**Figure 2:**
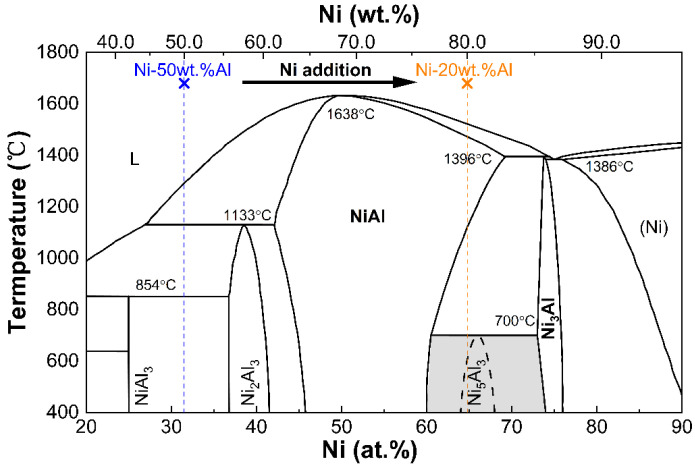
Ni-Al phase diagram showing the route to mix the Ni-50Al powder with an appropriate content of Ni powder to laser additively manufacture the β/γ’ two-phase Ni-Al intermetallic alloy.

The Ni-50Al powder should consist of Ni_2_Al_3_ and NiAl_3_, as indicated by the phase diagram presented in Figure 2 of the Ni-Al. Based on the binary phase diagram, when the Ni concentration of the Ni-50Al alloy is increased to a value (80 wt.% in this work as indicated by the yellow dashed line) within the range of 77 wt.% to 86 wt.%, it becomes possible to transform the Ni_2_Al_3_ and NiAl_3_ mixture into to a combination of NiAl and Ni_3_Al mixture. This transformation can be achieved by adding appropriate amounts of Ni into the Ni-50Al powder and alloying through LAM technology, which is a rapid non-equilibrium solidification process. The rapid solidification process of the laser molten pool derived from the mixture of the Ni-50Al and Ni powders would render the nucleation of the Ni_5_Al_3_ phase thermodynamically impossible. Consequently, this leads to the formation of β/γ’ Ni-Al dual-phase intermetallic alloy.

### 2.3. LAM Fabrication of β/γ’ Alloy without and with Seeding α-Al_2_O_3_ NPs

**Figure 3:**
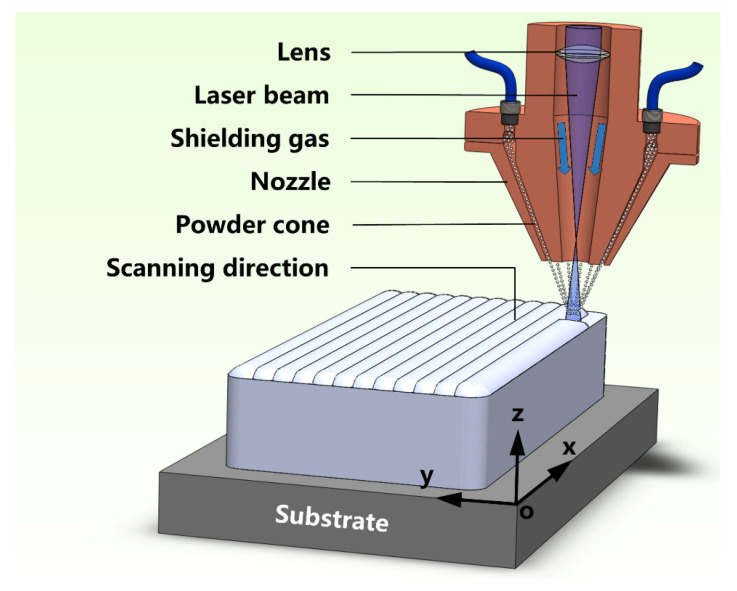
Schematic of the LAM method to manufacture the β/γ′ Ni-Al intermetallics in a cuboid shape.

After being dried by heating up to over 100 °C for 4 h, the Ni-50Al and Ni powder mixture were additively alloyed into the β/γ’ two-phase intermetallics through the use of the LAM method using an 8060 SYSTEM equipped with a Laserline LDF 3000-60 semiconductor laser equipment (RAYCHAM Inc., Nanjing, China) [39]. Figure 3 schematically illustrates the LAM process, during which the β/γ’ Ni-Al intermetallic alloy was scheduled to be manufactured onto a metal substrate. The metal substrate was heated up to 400 °C so as to prevent the generation of cracks in the alloy fabricated during the LAM process. The latter was carried out using a laser beam spot with a diameter of 1.5 mm under the conditions of 800 W laser power, 400 mm/min scanning speed with a hatch distance of 0.5 mm and 4.2 g/min powder feeding rate. Each deposition layer has a thickness of 0.8 mm. The as-printed alloys had a cuboid shape with dimensions of 10 mm × 10 mm × 3 mm. The as-deposited cubes were cut into small samples with dimensions of 8 mm × 8 mm × 1.5 mm. After being ground to 600 # SiC, part samples were further planted with α-Al_2_O_3_ NPs (ranging from 30 to 50 nm in size) through the use of LAM, which was processed using a laser beam spot of Φ3.0 mm, a scanning rate of 2000 mm/min, a hatch distance of 1.5 mm, and a laser power of 300 W.

### 2.4. Oxidation Test

The samples without and with the surface α-Al_2_O_3_ nano-seeds for oxidation were all cut from the top area of the as-deposited cube. Oxidation was carried out in air at 1000 °C at a heating rate of 50 °C/min using a SETARAM Setsys Evolution thermogravimetric analyzer (TGA, KEP technologies, Lyon, France). After oxidation, the samples were cooled to room temperature in the TGA.

### 2.5. Microstructural Characterization

The as-printed samples before and after oxidation were investigated through the use of a scanning electron microscope (SEM, FEI Inc., Hillsboro, OR, USA) equipped with an energy-dispersive X-ray spectrometer (EDS). The phase composition and microstructures of the samples were characterized using small-angle X-ray diffraction (XRD, Bruker Corporation, Billerica, Germany) and an optical microscope (OM, Olympus Corporation, Tokyo, Japan). In addition, the thermally grown alumina was characterized through the use of photostimulated–luminescence spectroscopy (PSLS, Zolix, Beijing, China), as reported in [40]. Argon laser (λ = 638 nm) excitation was used. The produced laser beam with a spot diameter of ~2 μm was focused through an optical microprobe onto the surface of the oxidized samples, and the luminescence spectra of the formed alumina were collected through the use of a CCD detector and were fitted and analyzed using commercial RTSSan 1.0 software.

## 3. Results and Discussion

### 3.1. Microstructure

**Figure 4:**
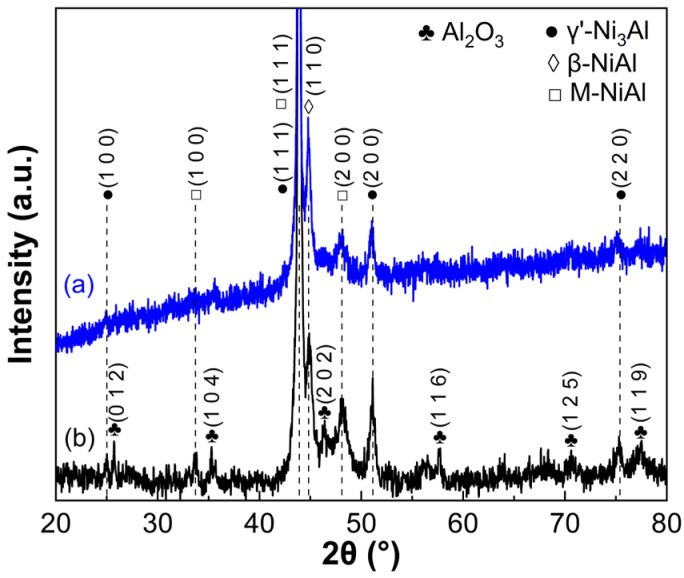
XRD pattern of the as-printed β/γ′ dual-phase intermetallic alloy (**a**) without and (**b**) with surface-seeded α-Al_2_O_3_ NPs.

The as-printed Ni-Al alloy contains 20 Al wt.% on the basis of EDS measurements. It is composed of β-NiAl, γ’-Ni_3_Al and minor M-NiAl phases, as presented in Figure 4. A similar observation was reported in our previous work [39]. The formation of M-NiAl can be attributed to the martensitic phase transformation that occurred during the rapid solidification of the molten laser pool. This phase was also observed in the NiAl alloys with Ni > ~77 wt.% (61 at.%), which resulted from rapid cooling from high temperatures [41,42]. In contrast, α-Al_2_O_3_ was identified through the use of XRD analysis from the as-printed β/γ’ Ni-Al intermetallic alloy integratedly added with α-Al_2_O_3_ NPs, indicative of successful planting of the oxide NPs onto the surface of the dual-phase alloy.

**Figure 5:**
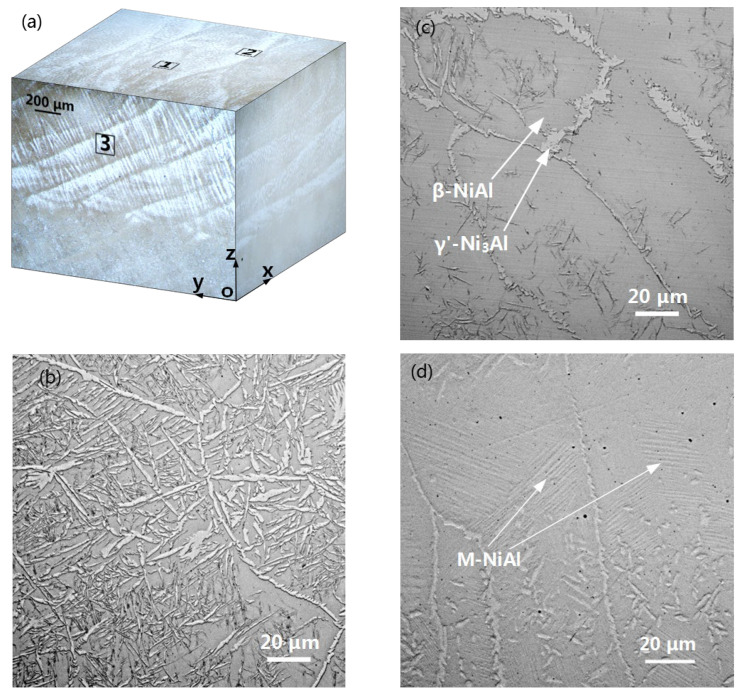
(**a**) The block diagram shows the OM microstructure of the LAM sample (its surface located on the XOY plane and close to the top surface of the as-printed cube). (**b**–**d**) OM morphologies at higher magnification of areas 1, 2 and 3 labeled in (**a**).

Figure 5a shows the three-dimensional OM microstructure of the LAM alloy cut from the area close to the top surface of the as-deposited cube. The XOY plane generally has two typical areas, with the framed areas numbered 1 and 2, respectively. Their magnified images show that area 1 was composed of a major β phase-dispersing high-density light dendritic precipitates of the γ’ phase (Figure 5b), while in area 2, the matrix β phase with scattered γ’ phase precipitation appeared (Figure 5c). Viewed from the magnified image of an area labeled 3 in the cross-sectioned YOZ plane (Figure 5d), M-NiAl, which exhibits a typical lath structure, as indicated by arrows, is observed. The different features of the as-printed β/γ’ two-phase intermetallic alloy undoubtedly resulted from the solidification process of the molten laser pool. The surface of the as-deposited cube was solidified at the fastest cooling rate, resulting in the rapid precipitation of the γ’ phase from the liquid phase. Consequently, a decrease in Ni concentration but an increase in Al concentration occurred in the deeper area of the molten pool. This, coupled with the relatively slower cooling rate there, made the γ’ phase nucleation a difficult process. The larger-sized γ’-free β phase matrix accordingly suffered martensitic phase transformation.

**Figure 6:**
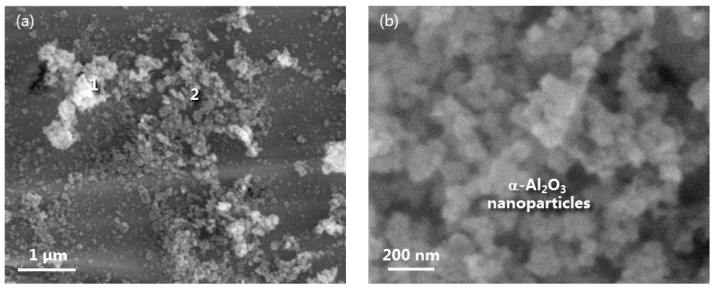
(**a**) Surface morphology of β/γ′ Ni-Al intermetallic alloy with the seeded α-Al_2_O_3_ NPs, (**b**) a higher magnification of area 2 labeled in (**a**).

Some as-printed samples were seeded with α-Al_2_O_3_ NPs via laser printing on their surface. Figure 6 shows the surface morphology of the β/γ’ two-phase intermetallic alloy dispersing the seeded α-Al_2_O_3_ NPs. The latter exhibited two different areas: minor area 1, where the α-Al_2_O_3_ NPs were agglomerated, and major area 2, where the oxide NPs were better distributed. In general, the α-Al_2_O_3_ NPs were loosely and discontinuously dispersed on the alloy surface. The β/γ’ two-phase alloys without and with the α-Al_2_O_3_ nano-dispersions were then oxidized for comparison.

### 3.2. Growth of Initial Alumina Phases

**Figure 7:**
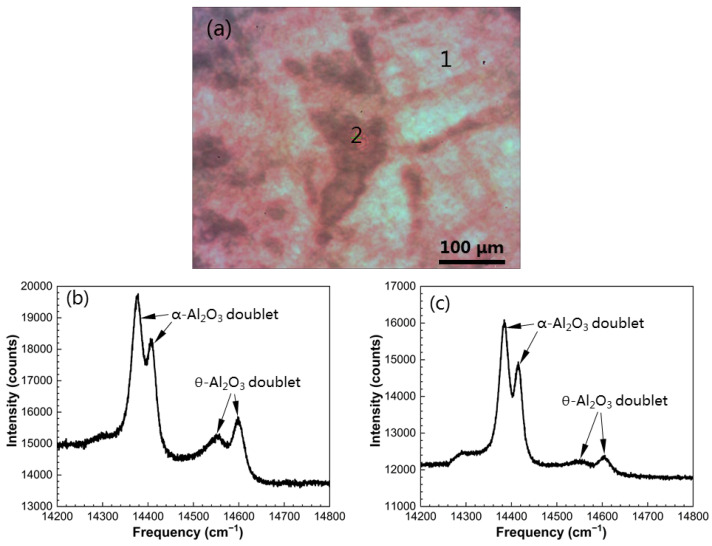
(**a**) Surface OM morphology of the LAM β/γ’ Ni-Al intermetallic alloy after 40 min oxidation in air at 1000 °C. (**b**,**c**) Luminescence spectrum acquired from regions 1 and 2 in (**a**), respectively.

**Figure 8:**
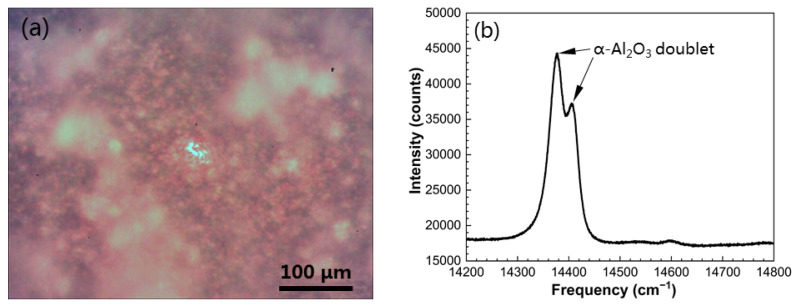
(**a**) Surface OM morphology of α-Al_2_O_3_ NP-seeded β/γ’ Ni-Al intermetallics after 40 min oxidation in air at 1000 °C. (**b**) Luminescence spectrum acquired by moving the objective lens across the sample surface.

After 40 min oxidation in air at 1000 °C, alumina scales formed on the β/γ’ intermetallic alloys without and with surface-seeded α-Al_2_O_3_ NPs were characterized through the use of PSLS. The naked two-phase alloy exhibited two distinct areas on the monitor with the luminescence spectrometer, the major β-NiAl growing “light” oxide (region 1) while the minor γ’-Ni_3_Al growing “darker” oxide (region 2), as depicted in Figure 7a. The representative PSLS of the two oxides is presented in Figure 7b,c, respectively. As is evident, α-Al_2_O_3_ doublets at 14,378.30 cm^−1^ and 14,405.54 cm^−1^ and θ-Al_2_O_3_ doublets at 14,553.81 cm^−1^ and 14,597.94 cm^−1^ occur on the β-NiAl. In comparison, the oxidized γ’ also displays two alumina polymorphs, but the θ-Al_2_O_3_ doublets have significantly weaker intensity. This suggests faster growth of α-Al_2_O_3_ on the γ’ phase with respect to the β phase. Figure 8a shows the surface morphology of the α-Al_2_O_3_ NPs-seeded two-phase alloys after the initial oxidation period. The two distinct areas showing different oxide mixtures mentioned above are no longer visible. By moving the objective lens back and forth across the sample surface, a typical luminescence spectrum was acquired, as shown in Figure 8b. The presence of θ-Al_2_O_3_ doublets is hardly seen, indicating that seeding α-Al_2_O_3_ NPs facilitates the thermal growth of α-Al_2_O_3_ from the beginning of oxidation.

### 3.3. Oxidation Kinetics

**Figure 9:**
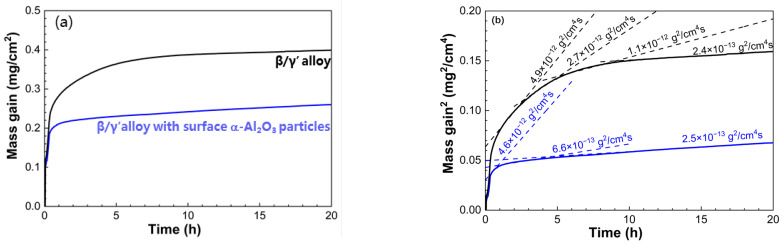
(**a**) Oxidation kinetics and (**b**) corresponding parabolic plots of the β/γ′ two-phase Ni-Al intermetallic alloys with and without surface α-Al_2_O_3_ NPs in air at 1000 °C.

Figure 9a displays the oxidation curves of the two β/γ’ two-phase intermetallic alloys for 20 h in air at 1000 °C. As is evident, seeding the α-Al_2_O_3_ NPs profoundly decreased the oxidation rate of β/γ’ intermetallic compounds. From the corresponding parabolic plots (Figure 9b), the β/γ’ two-phase intermetallic alloy experienced four stages with a decrease in the calculated oxidation parabolic constant (*k_p_*) from 4.9 × 10^−12^ g^2^/cm^4^·s in stage I, 2.7 × 10^−12^ g^2^/cm^4^·s in stage II, 1.1 × 10^−12^ g^2^/cm^4^·s in stage III down to 2.4 × 10^−13^ g^2^/cm^4^·s in stage IV. In contrast, the α-Al_2_O_3_ NP-seeded β/γ’ alloy quickly entered a steady-state oxidation period with *k_p_* of 2.5 × 10^−13^ g^2^/cm^4^·s after a very short initial stage with a *k_p_* of 4.6 × 10^−12^ g^2^/cm^4^·s. As will be demonstrated later, the oxidation of the β/γ’ two-phase alloy free of the α-Al_2_O_3_ particles was divided into four stages because the nickel aluminides preferentially grew θ-Al_2_O_3_. The I–III stages with decreased values of *k_p_* corresponded to the periods during which the growth of θ-Al_2_O_3_ along with its transformation to α phase occurred. The θ-to-α phase transformation took place at the θ-alumina/aluminide interface [28,35,36]. Compared to the first three stages, stage IV exhibited the oxidation rate with an order of magnitude lower *k_p_*, suggesting that a continuous α-Al_2_O_3_ layer was established at the interface. After being seeded with α-Al_2_O_3_ NPs, the β/γ’ alloy during the entire period was oxidized at a rate similar to that of the unseeded one in stage IV, indicative of the continuous α-Al_2_O_3_ layer formation almost from the very beginning.

### 3.4. Oxide Morphology Characteristics and α-Al_2_O_3_ NPs Effect on Oxidation

The oxidation curves indicate that seeding α-Al_2_O_3_ NPs on the surface of the β/γ’ intermetallic alloy almost one order of magnitude decreased the alloy’s oxidation rate. To clarify the effect of the α-Al_2_O_3_ nano-seeds on oxidation, the surface and cross-sectional morphologies of the oxide scales on the β/γ’ alloys without and with the oxide nano-seeds were investigated.

**Figure 10:**
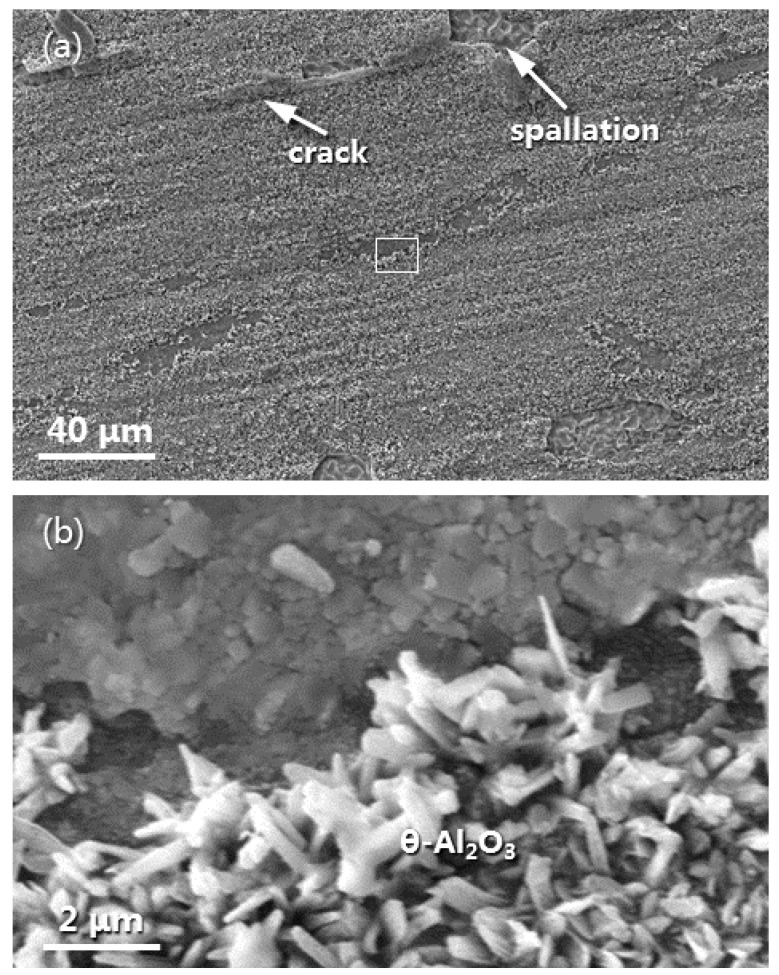
(**a**) Surface SEM morphology of the residual oxide scale formed on the LAM β/γ’ Ni-Al intermetallic alloy after 20 h oxidation in air at 1000 °C. (**b**) is a higher magnification of the framed area in (**a**).

Large-scale spallation of the oxide scale on the naked LAM β/γ’ alloy occurred after 20 h oxidation. Figure 10a shows the surface morphology of the residual oxide scale formed on the as-deposited dual-phase intermetallics for 20 h oxidation. Some cracks and spallation, as indicated by arrows, occurred. The thermally grown oxide on the other area, as framed, exhibited two different features. One was observed as needle- or rod-like oxide crystals, and the other appeared as granular-like oxide crystals, as clearly seen in Figure 10b at a higher magnification. Based on the PSLS results (Figure 7a), it can be inferred that needle- or rod-like oxide crystals represent θ-Al_2_O_3_ formed on the β-NiAl phase, while granular-like oxide crystals correspond to α-Al_2_O_3_ grown on the γ’-Ni_3_Al phase.

It has been extensively reported that thermally grown θ-Al_2_O_3_ crystals are in a meta-stable state and normally needle- or whisker-shaped. These crystals can undergo coarsening and blunting, finally transforming into rod-like α-Al_2_O_3_ crystals. This transformation process starts from the θ-Al_2_O_3_/alloy interface [27,28,40,43]. θ-Al_2_O_3_ preferential growth and transformation to α-Al_2_O_3_ has been observed during oxidation of β-NiAl at 1000 °C and below [12,27,28,29,30]. Strong θ-Al_2_O_3_ doublets were recorded through the use of PSLS from the β-NiAl phase of the bare two-phase alloy after 40 min oxidation, indicative of a preferential growth of θ-Al_2_O_3_ with the localized formation of α-Al_2_O_3_ beneath. In contrast, during the initial stage of oxidation, the γ’-Ni_3_Al phase of the bare two-phase primarily grew α-Al_2_O_3_ based on the PSLS analyses. This reason can be explained below.

During oxidation, γ’-Ni_3_Al normally grows NiO at first and then Al_2_O_3_ underneath. The diffusion reaction between the two oxides produces NiAl_2_O_4_ at the interface. Pérez et al. [44] reported that a Ni_3_Al powder metallurgical alloy formed relatively thicker NiO and NiAl_2_O_4_ layers above the inner Al_2_O_3_ layer during oxidation in air at high temperatures ranging from 930 °C to 1200 °C. However, an Al_2_O_3_-dominant scale, together with relatively much thinner NiO and NiAl_2_O_4_, was developed on a Ni_3_Al nanocrystalline alloy developed through the use of magnetron sputtering [45]. The result is understandable when following Wagner’s classic oxidation theory [46]. Al-selective oxidation benefits from a significant grain refinement of γ’-Ni_3_Al because the generated abundant grain boundaries dramatically enhance the diffusion flux of Al to the oxidation front. LAM is a non-equilibrium solidification process. The rapid solidification of γ’ would lead to grain refinement. Our recent work [see Supplementary Material] revealed that the grain size of γ’ phase in the LAM γ’/γ Ni-Al two-phase alloy decreased from ~250 nm to ~88 nm, with the Al concentration decrease from 22 at.% to 19 at.%. From this, it is proposed that during the oxidation of the γ’ phase in LAM dual-phase intermetallics, preferentially formed NiO quickly stops growing because it is undermined by a rapidly formed alumina layer. The PSLS result confirms the formation of an α-Al_2_O_3_-predominant scale on the γ’ phase during the early stage of oxidation (Figure 7c). This implies that the NiO formation on γ’, coupled with its grain refinement, promotes the rapid development of an α-Al_2_O_3_ layer, although further investigation is required to determine the underlying cause. However, as will be presented, the rapid development of an α-Al_2_O_3_ layer on the γ’ phase is in agreement with the cross-sectional observation of the naked LAM β/γ’ alloy after oxidation. The NiO crystals are hardly observed on the two-phase alloy after 20 h of oxidation because they have been swept over by the outward-growing part of the α-Al_2_O_3_ layer.

**Figure 11:**
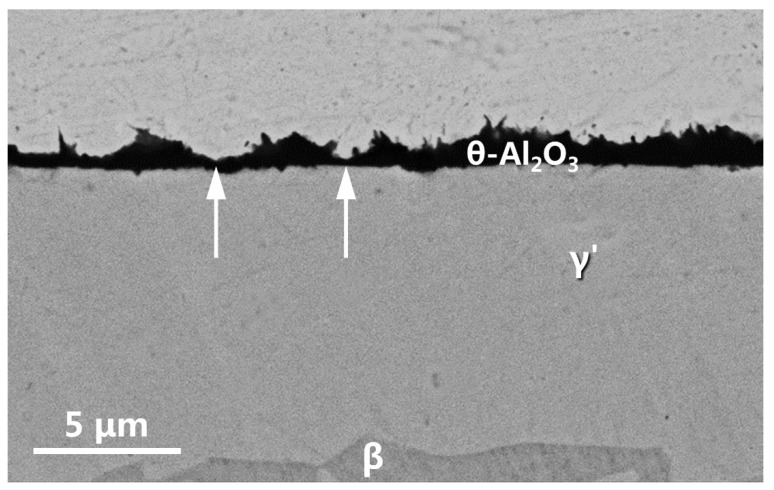
Cross-sectional SEM morphology of the residual oxide scale on the LAM β/γ’ Ni-Al intermetallic alloy after 20 h oxidation in air at 1000 °C.

Figure 11 shows the cross-sectional morphology of the residual alumina scale formed on the naked LAM β/γ’ alloy after 20 h of oxidation. The alumina scale was nonuniform in thickness, ranging from 0.6 μm to 2.1 μm, with a mean value of ~1.1 μm. The needle-shaped θ-Al_2_O_3_ crystals, which were typically observed, grew on the original β phase. In addition, as addressed above, the γ’ phase exhibited growth of α-Al_2_O_3_. However, due to its naturally lower thickening rate compared to neighboring θ-Al_2_O_3_, the oxide scale formed there (as indicated by arrows) appeared thinner than the θ-Al_2_O_3_ scale formed on the β phase. Moreover, the Al consumption by oxidation led to the complete degradation of the high Al-containing β phase at a depth of ~10 μm to the “light” phase. It was actually the γ’ phase due to the EDS acquisition of ~73.3 at.% Ni and 26.7 at.% Al there.

The transformation of θ-Al_2_O_3_ to α-Al_2_O_3_ is known to result in a 10 vol.% shrinkage [32,33,34,47]. In the LAM two-phase alloy, the β-NiAl phase thermally grows θ-Al_2_O_3_ with a significantly increased volume fraction compared to the γ’-Ni_3_Al phase. The tensile stresses induced by the θ-to-α transformation, combined with the inherently “harder” nature of the β phase with respect to the γ’ phase, likely initiate micro-crack generation in the alumina scale and, consequently, alumina spallation. Extensive results have been reported on the spallation of the alumina scale formed on nickel aluminides [29,31,32,33,34]. In contrast, no spallation was seen after oxidation for the LAM β/γ’ Ni-Al alloy integratedly seeded with α-Al_2_O_3_ NPs. This can be attributed to the negligible stress caused by the alumina phase transformation in the alumina scale formed on the α-Al_2_O_3_ NPs-seeded β/γ’ alloy due to its enhanced ability to form an α-Al_2_O_3_ scale during the early stages of oxidation (Figure 8).

**Figure 12:**
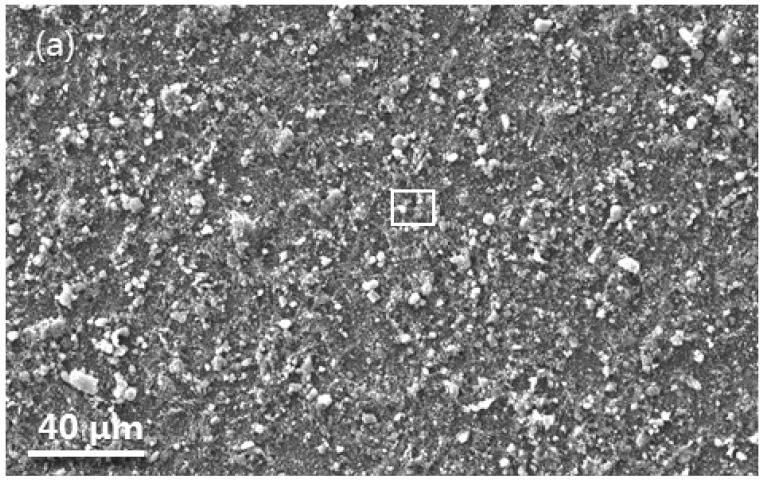

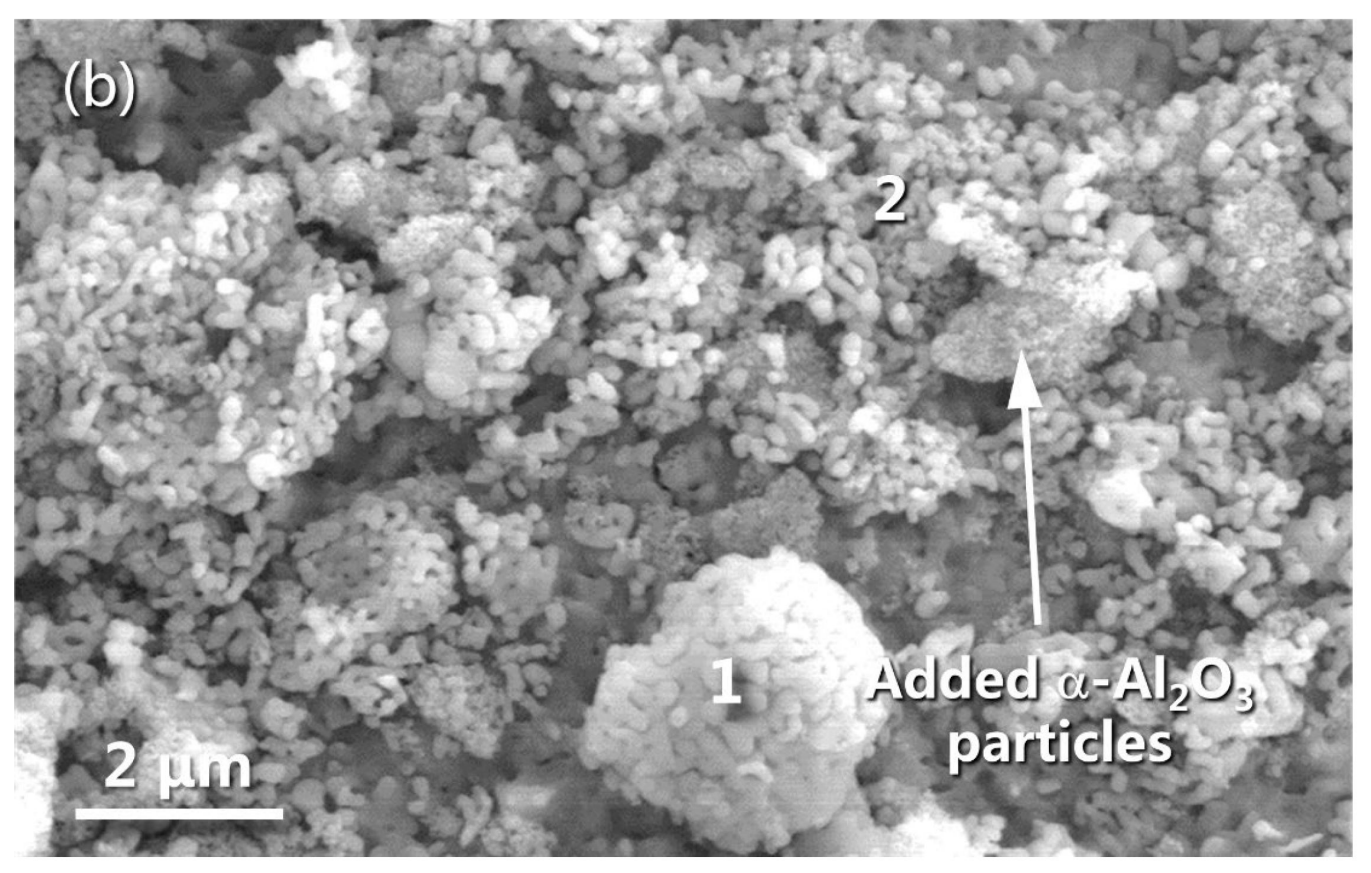
(**a**) Surface SEM morphology of the LAN β/γ’ Ni-Al intermetallics integratedly seeded with surface α-Al_2_O_3_ NPs for 20 h oxidation in air at 1000 °C. (**b**) is a higher magnification of the framed area in (**a**).

**Figure 13:**
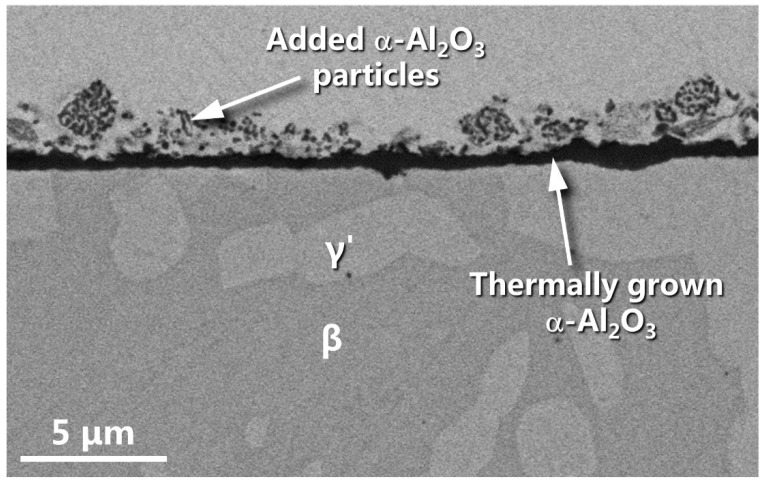
Cross-sectional SEM morphology of the LAM integrated β/γ’ Ni-Al intermetallic alloy seeded with surface α-Al_2_O_3_ NPs for 20 h oxidation in air at 1000 °C.

Figure 12a shows the surface feature of the LAM β/γ’ intermetallic alloy for 20 h oxidation. Viewed at higher magnification, the surface generally displayed two features as marked with 1 and 2 in Figure 12b. Their topographic characteristics corresponded well to the α-Al_2_O_3_ NPs agglomerates and dispersoids that were originally seeded (Figure 6a). The difference is that α-Al_2_O_3_ NPs became somewhat larger in size due to their growth as a result of sintering during oxidation. Beneath the seeded α-Al_2_O_3_ NPs appeared on an alumina scale, as seen in Figure 13. There were two major findings. First, below the alumina scale only appeared a thin Al-depleted band (<1 μm in thickness) and below the band remained a considerable area fraction of the β phase, possibly due to the slower oxidation of the α-Al_2_O_3_ NP-seeded dual-phase intermetallics than the naked counterpart. Second, the alumina scale was uniform in thickness, with a mean value of ~0.6 μm. It was much thinner than the alumina scale formed on the naked β/γ’ alloy. The result is consistent with the PSLS characterization that seeding the surface α-Al_2_O_3_ NPs promoted the thermal growth of α-Al_2_O_3_ almost from the onset of oxidation on the entire surface of dual-phase intermetallics, including the seeded β phase. The growth rate of α-Al_2_O_3_ is one or two orders of magnitude slower than that of θ-Al_2_O_3_ [29]. Consequently, the oxidation rate of the α-Al_2_O_3_ NP_-_seeded alloy compared to the bare one is significantly reduced after the establishment of a continuous α-Al_2_O_3_ scale (Figure 9). The slower growth of the α-Al_2_O_3_ scale on the α-Al_2_O_3_ NPs-seeded alloy is beneficial in terms of preventing the formation of a large-sized cavity at the alumina scale/nickel aluminide interface [35,36]. This would be another contributing factor involved in the formation of a more adherent alumina scale on the α-Al_2_O_3_ NP_-_seeded β/γ’ intermetallic alloy with respect to the naked counterpart.

Based on the aforementioned results and interpretations, the enhanced oxidation resistance of the LAM β/γ’ dual-phase alloy can be attributed to the promotion of the thermal growth of α-Al_2_O_3_, which is fundamentally induced by surface-seeded α-Al_2_O_3_ NPs. Peng et al. [48] proposed that θ-Al_2_O_3_ rather than α-Al_2_O_3_ preferentially grows on M_x_Al_y_ (M = Ni, Fe, Co) such as β-NiAl due to the fact that a higher energy barrier is needed for oxidation through the route of α-Al_2_O_3_ direct growth in comparison to the conventionally observed route of θ-Al_2_O_3_ preferential growth and subsequent transformation to α-Al_2_O_3_ through a “synchro-shear” mechanism [48]. When M_x_Al_y_ is dispersed with NPs of metal oxide with either a *hcp* structure or a *hcp* O sublattice structure, these oxide NPs would exert a “template” effect, catalyzing the direct growth of corundum α-Al_2_O_3_. Taking TiO_2_ as an example [43], the rutile oxide and α-Al_2_O_3_ share the well-matching O sublattice. Thus, the α-Al_2_O_3_ stable embryo can be energetically allowable to form around the surface TiO_2_ NPs without the necessity of shear displacement but just simply through the layer-by-layer stacking of O anions following the hcp O sublattice structure of TiO_2_ on the energy-favorable crystalline planes and rearrangement of Al^3+^ at empty honeycomb octahedral interstices sites (O_h_) to form a “honey comb” lattice structure via cation diffusion. The α-Al_2_O_3_ embryos occur at the point of contact between TiO_2_ NPs and the aluminide. The oxide embryos then grow in size around the NPs and finally fill the spacings among them. This results in dispersing the NPs of metal oxide with a *hcp* or *hcp* O sublattice structure onto to nickel aluminide, catalyzing the thermal growth of α-Al_2_O_3_ NPs, as has been reported in our previous works [35,36,43].

## 4. Conclusions

A Ni-Al intermetallic alloy consisting of β-NiAl with poor strength and γ’-Ni_3_Al with better toughness, along with the surface seeding α-Al_2_O_3_ NPs, were integratedly developed through the use of LAM. During oxidation in air at 1000 °C, the naked β/γ’ alloy formed an alumina scale with a nonuniform thickness. Thick θ-Al_2_O_3_ grew on the β phase, while thinner α-Al_2_O_3_ primarily occurred on the γ’ phase. Compared to the bare β/γ’ alloy, the α-Al_2_O_3_ NP-seeded counterpart exhibited almost coverage of an α-Al_2_O_3_ scale during the early stage of oxidation, resulting in the formation of a thinner and uniformly thick alumina scale during long-term oxidation. It is proposed that the seeded α-Al_2_O_3_ NPs acted as crystallographic templates, which energetically catalyzed the thermal growth of α-Al_2_O_3_ not only on the β phase but also on the γ’ phase. The surface seeding α-Al_2_O_3_ NPs also helped the two-phase intermetallic alloy to develop a more adherent α-Al_2_O_3_ scale. The result suggests that LAM would be a promising technique for the integrated development of Ni-Al intermetallic alloys with better comprehensive mechanical properties and high-temperature oxidation resistance.

## Data Availability

The raw/processed data required to reproduce these findings cannot be shared at this time due to technical or time limitations. They will be shared upon request.

## Supplementary Materials

The following supporting information can be downloaded at: https://www.mdpi.com/article/10.3390/ma16227205/s1, Figure S1: Surface SEM morphologies of LMD γ’/γ (a) Ni-22Al and (b) Ni-19Al.
