# Supplementary material for: Integrated Laser Additive Manufacturing of α-Al2O3 Nanoparticle-Seeded β/γ’ Ni-Al Intermetallic Alloy with Enhanced High-Temperature Oxidation Performance
Source: Materials (Basel). 2023 Nov 17;16(22):7205. doi: 10.3390/ma16227205 (PMC10673039; doi:10.3390/ma16227205)
Supplement: Supplementary file 1 [file materials-16-07205-s001.zip › materials-2679996-supplementary.pdf]

**Supplementary material**

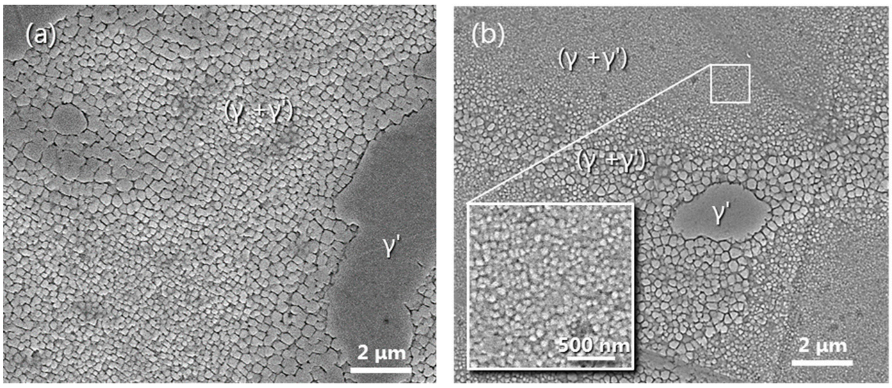


**Figure S1.** Surface SEM morphologies of LMD γ'/γ (a) Ni-22Al and (b) Ni-19Al are presented in figures above, respectively. The result shows that the non-equilibrium solidification process of LAM method would lead to a grain refinement of γ' phase, which decreased down from ~250 nm to ~88 nm with the Al concentration decrease from 22 at. % to 19 at. %. This result has been mentioned and discussed in page 9 of the revised manuscript.
